# Notices for May

**Published:** 1868-05

**Authors:** 


### NOTICES FOR MAY.

HARPER & BROTHERS have published a Manual of Physical Exercises, 316 pages, 12 mo., comprising Gymnastics, Rowing, Skating, Fencing, Cricket, Calisthenics, Sailing, Swimming, Sparring, Base-Ball, together with rules for training and sanitary suggestions, by William Wood, Instructor in Physical Education, with 125 illustrations. Chapt. 22, which is the last, is a record of the best time ever made in the various forms of locomotion, from the walking of a man to the speed of a cannon ball, thus the best walking was half a mile in three minutes and eighteen seconds; the best running, a hundred yards in nine seconds; cannon ball a thousand miles an hour. The book is very suggestive, entertaining, practical and instructive.

THE AMERICAN TRACT SOCIETY, 150 Nassau St., New York; 40 Cornhill, Boston; Rochester, 75 State St.; Philadelphia, 1210 Chestnut St.; Baltimore, 70 West Fayette St.; Cincinnati, 163 Walnut St.; Chicago, 7 Custom House Place; St. Louis, 19 South Fifth St., have issued "Companion to the Bible, Part 1st, Evidences of Revealed Religion, by Rev. E. P. Barrows, D. D., Professor of Biblical Theology, in paper cover. This most important work is to be continued.

ATLANTIC MONTHLY, contains a number of very readable articles, but the more discriminating and more wary religious papers keep a sharp watch over it. One of them is pretty out-spoken, in saying "Oliver Wendell Holmes' infidelity continues to poison the youth of our best families in the 'Atlantic.' Charles Hodge's article may sugar-coat the poison somewhat, but the cheat is unworthy the usual frankness of Messrs. Ticknor & Fields." Is it right for any publisher to allow any article in his periodical which disparages, even indirectly, the religious sentiment of its readers? We think it is not right, but an impudent fraud, and the question comes practically home to every Christian parent, ought I to admit into my family a publication which, in the main, is admirably conducted, but which, every now and then, gives religion a sly dig under the fifth rib; the very excellency of the publication in general, gives its occasional poison an aggravated malignity, and it is to be hoped that the publishers of the "Atlantic" will follow a better path, knowing that they are accountable to society, and will be held strictly so at the judgment day.

THE BOSTON MEDICAL AND SURGICAL JOURNAL comes to us greatly enlarged, and is now of the size of the "London Lancet," indicating, as it merits, increased prosperity. Price, $4 a year in advance, in either weekly or monthly numbers.

PHRENOLOGICAL JOURNAL, published monthly by S. R. Wells, 389 Broadway, New York, $3, contains a great deal of interesting and practical matter. No. 4, of Vol. 47 has portraits and characters of Adelina Patti, Dr. Isaac Jennings, Allen A. Griffeth, the Western Elocutionist, Charles 1st of Fngland, King and Queen of Greece, Revs. M. J. Raphel, S. M. Isaacs, Isaac Leeser, A. De Sola, Dr. Adler, Dr. Illowy, and six other prominent Jewish divines. The Family, by Mrs. E. O. Smith, Inordinate Affection, Dissipation, Disease, &c. Single Nos. 30 cents.

THE GOSPEL AMONG THE ANIMALS; OR, CHRIST WITH THE CATTLE, by Sam'l Osgood, D. D., a plea for a greater consideration of the comfort of domestic animals, or the true relation of man to the animal world. "A merciful man is merciful to his beast;" "That mercy I to others show, that mercy show to me," are suggestive themes, and merit our practical attention.

COLTON'S JOURNAL OF GEOGRAPHY AND COLLATERAL SCIENCES, being a record of discovery, exploration and survey, issued quarterly from Colton's Geographical Establishment, 172 William street, New York, $1 a year. To teachers, clergymen, and every family of intelligence and cultivation, this publication is highly instructive, and is commended to their notice.

THE SOUTHERN PRESBYTERIAN REVIEW, conducted by an association of ministers, in Columbia, South Carolina, is published quarterly, at $3 a year; single numbers, $1. All communications should be addressed to Rev. James Woodrow, Columbia, S. C. "Right and Wrong," by Rev. F. A. Ross, D. D., being a review of "Faith in God's Word," by Rev. Albert

Barnes ; "Value of the Christian Pulpit," by Rev. R. Q. Mallard ; "The Church and Politics," by B. T. W. ; "The Tishbite and the Baptist ;" "What is Conscience," by Rev. Henry M. Smith ; critical notices of "Trench's Study of the Gospels;" Scott's " Creed of the Apostles ;" Shedd's " Homiletics." This periodical merits the patronage of the "Presbyterian Family;" it it written for by able, christian men, who have been long known to the church, and who have her confidence.

" ONE OF THE CHOSEN," is a beautiful little print, just published by Bradley & Co., 66 North 4th St., Philadelphia, Penn., and is worthy of a place on the wall of our children's chambers. There are children whose blossom-like lives unclose, whose sweetness thrills us, and whose tender, serious, wistful ways, fill us with vague forbodings, who faint and fade and die. Such a child has caught the artist's eye, and is here given as one of "The Chosen Ones" in Heaven.

LONDON LANCET, $5 a year, published monthly by W. C. Hazard, 32 Peekman St., New York. By an arrangement with the London publishers, the "Lancet" will appear in New York and London on the 1st of each month. Number two of the volume for 1868, contains a large amount of the latest medical intelligence, and it ought to be on the table of every medical practitioner who aims to keep up with the times in medical and surgical observation, practice and progress.

POPULAR EDUCATION, being an Address of Edward Shippen, Esq., a distinguished and able lawyer of Philadelphia, delivered on the occasion of the dedication of the "Hollingsworth School," and published at the earnest solicitation of many prominent citizens, and well deserves to be placed in the libraries of the country for future reference, as it embodies many facts which should be kept in remembrance by all who think as William Penn, "Good Instruction is better than Riches." In this connection we take pleasure in noticing the annual report of W. R. White, Esq., General Superintendent of Free Schools, for the year 1867, of West Virginia. The report itself indicates great industry, system and ability in collecting and presenting such a large collection of facts from all parts of the

State, and the interest which the people in general, as well as the legislative bodies, have manifested in the cause of popular education, is worthy of all praise, and it is to be hoped that Mr. White may long be continued in the very responsible position which he now occupies.

"THE GALAXY," for April, announces that editorial communications must be addressed "To the Editor."

